# Influenza vaccination during pregnancy and influencing factors in Korea: A multicenter questionnaire study of pregnant women and obstetrics and gynecology doctors

**DOI:** 10.1186/s12884-021-03984-2

**Published:** 2021-07-16

**Authors:** Byung Soo Kang, San Ha Lee, Woo Jeng Kim, Jeong Ha Wie, In Yang Park, Hyun Sun Ko

**Affiliations:** 1 Department of Obstetrics and Gynecology, Seoul St. Mary’s Hospital, College of Medicine, The Catholic University of Korea, Seoul, Republic of Korea; 2 Department of Obstetrics and Gynecology, Eunpyeong St. Mary’s Hospital, College of Medicine, The Catholic University of Korea, Seoul, Republic of Korea

**Keywords:** Influenza, Maternal, Vaccination

## Abstract

**Background:**

Although the World Health Organization and health authorities in most countries recommend that pregnant women receive inactivated influenza virus vaccines, coverage remains low. This study aimed to investigate (1) the proportion of pregnant women who received an influenza vaccination and influencing factors and (2) the proportion of obstetrics and gynecology (OBGYN) doctors who routinely recommend influenza vaccination to pregnant women and influencing factors.

**Methods:**

Two separate, anonymized questionnaires were developed for physicians and pregnant and postpartum women and were distributed to multicenters and clinics in South Korea. The proportions of women who received influenza vaccination during pregnancy and OBGYN doctors who routinely recommend the influenza vaccine to pregnant women were analyzed. Independent influencing factors for both maternal influenza vaccination and OBGYN doctors’ routine recommendations to pregnant women were analyzed using log-binomial regression analysis.

**Results:**

The proportion of self-reported influenza vaccination during pregnancy among 522 women was 63.2%. Pregnancy-related independent factors influencing maternal influenza vaccination were “(ever) received information about influenza vaccination during pregnancy” (OR 8.9, 95% CI 4.17–19.01), “received vaccine information about from OBGYN doctors” (OR 11.44, 95% CI 5.46–24.00), “information obtained from other sources” (OR 4.38, 95% CI 2.01–9.55), and “second/third trimester” (OR 2.41, 95% CI 1.21–4.82)..

Among 372 OBGYN doctors, 76.9% routinely recommended vaccination for pregnant women. Independent factors effecting routine recommendation were “working at a private clinic or hospital” (OR 5.33, 95% CI 2.44–11.65), “awareness of KCDC guidelines” (OR 3.11, 95% CI 1.11–8.73), and “awareness of the 2019 national free influenza vaccination program for pregnant women” (OR 4.88, 95% CI 2.34–10.17). OBGYN doctors most commonly chose ‘guidelines proposed by the government or public health (108, 46%) and academic committees (59, 25%), as a factor which expect to affect the future recommendation

**Conclusion:**

This study showed that providing information about maternal influenza vaccination, especially by OBGYN doctors, is crucial for increasing vaccination coverage in pregnant women. Closer cooperation between the government and OBGYN academic societies to educate OBGYN doctors might enhance routine recommendations.

**Supplementary Information:**

The online version contains supplementary material available at 10.1186/s12884-021-03984-2.

## Background

Compared with the general population, pregnant women are at high risk for influenza-related complications due to their altered immunity and increased cardiopulmonary burden [1, 2]. During the 2009 H1N1 influenza pandemic season, maternal influenza infection was associated with severe complications resulting in maternal death and admission to intensive care units [3]. Additionally, influenza vaccines are not licensed for use in infants younger than six months. Therefore, infants younger than six months cannot be protected except through maternal immunization. According to global statistics, approximately 228,000 (95% Confidence Interval (CI) 150,000–344,000) of annual hospitalizations of infants younger than six months were associated with influenza [4]. Furthermore, infants younger than six months with confirmed influenza infection were at highest risk for hospitalization due to neurologic or pulmonary complications and, thus, for admission to an intensive care unit [5, 6]. Several studies have demonstrated the effectiveness of maternal influenza vaccination for protecting infants younger than six months from influenza, respiratory infection, and severe pneumonia [7, 8].

Although the World Health Organization (WHO) and health authorities in most countries recommend pregnant women receive a vaccination with inactivated influenza virus [9–11], vaccination coverage is not sufficient. Influenza vaccination rates in people aged 65 years or older, which is an indicator of annual influenza vaccination in the Organization for Economic Cooperation and Development (OECD) countries, were less than 60% in most countries, although it was over 80% in Korea [12]. Since 2012, the Korean Centers for Disease Control and Prevention (KCDC) has recommended vaccinating pregnant women as well as women contemplating pregnancy during the flu season under the Guidelines of Vaccination for Adults [13]. However, it has been reported that the vaccination rate associated with pregnancy was less than 40% [14, 15]. A national free immunization program for influenza was developed for pregnant women to increase vaccine coverage during the 2019–2020 flu season in Korea. This study aimed to investigate 1) the proportion of pregnant women who received an influenza vaccination and influencing factors and 2) the proportion of obstetrics and gynecology (OBGYN) doctors who routinely recommend influenza vaccination for pregnant women and their influencing factors.

## Methods

### Study population and recruitment

The survey was conducted from October 15, 2019, to December 31, 2019. The questionnaires for pregnant women were completed during routine antenatal care visits at a mix of 30 public and private clinics or hospitals located in metropolitan areas and the eight provinces of South Korea. Medical doctors or trained assistants distributed either a paper form or an online link to the survey in Google forms in person via opportunistic sampling at antenatal clinics or wards. Questionnaires for physicians were distributed to medical doctors registered with the Korean Society of Maternal–Fetal Medicine (KSMFM), Korean Society of Perinatal Medicine (KSPM), Korean Association of Obstetrics and Gynecology (KAOG), or Korean Society of Ultrasound in Obstetrics and Gynecology (KSUOG) via a paper form or an online link to the survey in Google forms by e-mail. Participation in this survey was voluntary, and no financial or other incentives were offered. Response to the survey implied consent. Information provided by the participants was voluntary and possibly incomplete.

### Survey questionnaires

Two questionnaires were developed and used anonymously to survey pregnant or postpartum women and OBGYN doctors (Suppl. 1 & 2). The questionnaires were adapted from previously self-administered questionnaires [16–18] composed by a multidisciplinary study team that included OBGYN doctors, biomedical statisticians, and pregnant women. A pilot survey involving both target groups was conducted to ensure questionnaire comprehensiveness. Because some participants did not know that the national free influenza vaccination program in the 2019–2020 flu season included pregnant women, we added the response option “free vaccination program” to question 10–2 in the questionnaire.

The questionnaire for pregnant or postpartum women assessed the following characteristics: age, pregnancy duration, parity, natural conception or use of assisted reproduction, education level, occupation, and administrative district of residential areas. Inclusion criteria were pregnant women over six weeks’ gestation with confirmed fetal heartbeat by ultrasonography and postpartum women within six weeks after delivery. The questionnaire for physicians queried the following characteristics: age, sex, recent maternity care, and characteristics of their employer, including whether their clinic or hospital is private or public and within which administrative district it is located. Residential areas and physicians’ work locations were divided into metropolitan and non-metropolitan areas. Metropolitan areas included Seoul, Busan, Gwangju, Incheon, Ulsan, Daejeon, Sejong, and Kyunggi provinces around Seoul. Non-metropolitan areas included Chuncheon, Gyeongsang, Jeolla, Gangwon, and Jeju provinces.

Pregnant and postpartum women were asked the following: (1) whether they received influenza vaccination during pregnancy in the 2019–2020 flu season; (2) whether they received information about influenza vaccination; (3) information sources; (4) influenza vaccination during a previous pregnancy; (5) reasons for not receiving influenza vaccine; and (6) factors influencing future vaccination. The women were classified according to self-reported influenza vaccination status for the flu season of 2019–2020. Response options for questions (1), (2), and (4) were yes or no. Questions about information sources permitted multiple responses. If a response included OBGYN doctors with or without other sources, it was designated to “OBGYN doctors.” If a response included other sources such as public health, media, friends, or family but not OBGYN doctors, the source was designated to “other sources.”

OBGYN doctors were grouped according to whether they provided routine recommendations for the influenza vaccine for pregnant women based on an affirmative response to the question, “Do you recommend the influenza vaccine to pregnant women in your clinic?”. OBGYN doctors who answered, “always recommend vaccination,” were designated to the, “routine recommendation group,” and OBGYN doctors who responded, “sometimes or never recommended vaccination,” were designated to the, “passive recommendation group.” Physician awareness of the 2019 national free influenza vaccination program for pregnant women and government recommendations such as “all pregnant or breastfeeding women during flu season are primarily recommended to receive an inactive influenza vaccine” was evaluated. Attitudes toward providing information about influenza vaccination for pregnant women were analyzed based on responses to the following questions: 1) “Do you provide information about influenza vaccination to pregnant women?” and 2) “Do you recommend influenza vaccination during pregnancy?”.

Also, the survey sought to determine each physician’s own influenza vaccination status during the previous flu season. Physicians were asked about influencing factors for future recommendations for influenza vaccination for pregnant women. Ethical approval was granted by the Institutional Review Board of The Catholic University of Korea (KC19QES10646).

### Sample size calculation

The sample size for the survey of pregnant or postpartum women was calculated with the following assumptions: the proportion of women having received the influenza vaccine during pregnancy was 50%, with a confidence interval of 95% and an alpha of 0.05. The initial calculated minimum sample size was 384 participants. Given the nonresponse rate (10%) and the incomplete responses rate (30%), however, 538 pregnant women were recruited to meet the minimum sample size. The sample size for the survey of OBGYN doctors was calculated based on the estimation that 60% of OBGYN doctors routinely recommend influenza vaccination to pregnant women, with a 95% confidence interval and an alpha of 0.05. Therefore, the minimum number of required OBGYN doctors was estimated at 360. Given the 55% response rate, however, the minimum number of participants required was estimated as 640. Data collection was stopped when the minimum numbers of responses for the analyses were reached.

### Data analysis

We performed all data analyses using SPSS (version 24.0; SPSS Inc., Chicago, IL, USA). Continuous variables of age of respondents and gestational weeks of pregnant women were presented as mean ± standard deviation and compared using Student’s t-test. All other variables were categorical data, which were expressed as number (%) and compared using the Chi-square test. To assess influencing factors associated with vaccine uptake by pregnant women, we calculated the odds ratios (ORs) and 95% CIs using log-binomial regression models. Variables with a significant cutoff, p < 0.2, between vaccinated and unvaccinated groups in univariate analyses were included in multivariate analyses, after adjustment for maternal age, residence, education, and occupation. Univariate analysis identified variables (p < 0.2) with OBGYN doctors’ routine recommendations for influenza vaccination between the routine and passive recommendation groups. After adjustment for physician age, sex, and location of clinic or hospital, significant variables were identified in multivariate analyses. Statistical significance was set at p < 0.05.

## Results

### Demographic characteristics of pregnant women

**Fig. 1:**
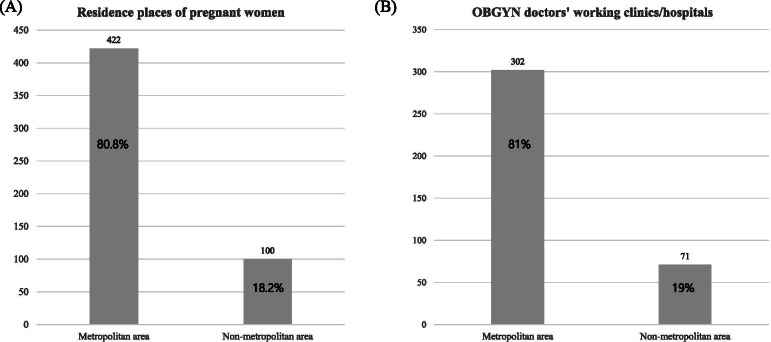
Distributions of respondents. (**A**) Residences of pregnant women (*n* = 522) (**B**) OBGYN^a^ doctors working clinics/hospitals (*n* = 383). ^a^OBGYN: obstetrics and gynecology. This file ‘South Korea location map’ by NordNordWest in Supplement 3 for presenting metropolitin and non-metropolitan area is licensed under CC BY-SA 3.0 < https://creativecommons.org/licenses/by-sa/3.0 > , via Wikimedia Commons.

A total of 522 questionnaires was eligible to be analyzed after excluding 34 incomplete questionnaires. The questionnaires were collected online (10.2%, 53/522) or via paper survey (89.8%, 487/522). The residential distribution of pregnant (n = 492) and postpartum women (n = 30) is presented in Fig. 1(A) and Suppl. 3, which indicate that 80.8% of respondents lived in a metropolitan area.

The demographic characteristics associated with influenza vaccination are presented in Table 1. There were significant differences in gestational age and pregnancy period between the two groups (p = 0.002, for both). Women in the vaccinated group were significantly better informed about the influenza vaccine than were women in the unvaccinated group (p < 0.001). Additionally, women in the vaccinated group were more likely to be informed about the vaccine by their OBGYN doctor than were women in the unvaccinated group (p < 0.001). There were no significant differences in mean maternal age, parity, conception method, education, metropolitan residence, occupation, or influenza vaccination during the previous pregnancy.

**Table 1:** Demographic characteristics of pregnant or postpartum women

Characteristics	Vaccinated (*n* = 330)	Unvaccinated (*n* = 192)	*p*-value
Maternal age (yrs) (mean ± SD)	33.32 ± 3.85	33.18 ± 4.53	0.069
Gestational age (weeks) (mean ± SD)	31.28 ± 20.27	26.31 ± 9.12	0.002
Pregnancy period			0.002
≤ 13 weeks (n, %)	17 (5.2)	25 (13.0)	
14–27 weeks (n, %)	67 (20.3)	45 (23.4)	
≥ 28 weeks (n, %)	230 (69.7)	108 (56.3)	
Postpartum (n, %)	16 (4.8)	14 (7.3)	
Nulliparous women (n, %)	219 (66.4)	122 (63.5)	0.514
Assisted reproduction (n, %)	29 (6.6)	19 (9.9)	0.790
Education (college degree or higher) (n, %)	304 (92.1)	168 (87.5)	0.084
Metropolitan residence (n, %)	270 (81.8)	15 (79.2)	0.458
Occupation			0.186
Housewife (n, %)	145 (43.9)	73 (38.0)	
Other than housewife (n, %)	185 (56.1)	119 (62.0)	
Ever received information about influenza vaccination during pregnancy (n, %)	321 (97.3)	155 (80.7)	< 0.001
Source of information			< 0.001
OBGYN^a^ doctor (n, %)	239 (72.4)	83 (43.2)	
Non-OBGYN doctors, public health care, media, friends, or family (n, %)	91 (27.6)	109 (56.8)	
Influenza vaccination in a previous pregnancy (n, %)^b^	71 (64.5)	34 (54.8)	0.205

^a^OBGYN, obstetrics and gynecology; ^b^responses from parous women

Values are presented as mean ± standard deviation or n (%)

### Influencing factors for maternal influenza vaccination

In univariate analyses, “ever received information about influenza vaccination during pregnancy,” “received vaccine information from OBGYN doctor or other sources,” and “second/third trimester” were significantly associated with maternal influenza vaccination (Table 2). In multivariate analyses adjusted for maternal age, education, occupation, location of residence, and pregnancy period, “ever received information about influenza vaccination during pregnancy” (OR 8.9, 95% CI 4.17–19.01), “received vaccine information about from OBGYN doctors” (OR 11.44, 95% CI 5.46–24.00), “information obtained from other sources” (OR 4.38, 95% CI 2.01–9.55), and “second/third trimester” (OR 2.41, 95% CI 1.21–4.82) significantly increased the odds for influenza vaccination.

**Table 2:** Associated factors for maternal influenza vaccination among pregnant or postpartum women

Factors	Univariate			Multivariate		
	**Crude OR**^**a**^	**95% CI**^**b**^	**p-value**	**Adjusted OR**	**95% CI**	***p*-value**
**Ever received information about influenza vaccination**						
No	1			1		
Yes	8.51	4.01–18.08	< 0.001	8.9^**c**^	4.17–19.01	< 0.001
**Source of information**						
No	1			1		
Non-OBGYN^d^ doctors, public health care, media, friends, and family	4.37	2.02–9.44	< 0.001	4.38^c^	2.01–9.55	< 0.001
OBGYN doctor	11.45	5.49–23.87	< 0.001	11.44^c^	5.46–24.00	< 0.001
**Pregnancy period**						
1^st^ trimester	1			1		
2^nd^ or 3^rd^ trimester or postpartum	2.76	1.45–5.25	0.002	2.41^e^	1.21–4.82	0.013
**Influenza vaccination in previous pregnancy**						
No	1					
Yes	1.25	0.80–1.96	0.321			

^a^OR, odds ratio; ^b^CI, confidence interval

^c^Adjusted for maternal age, education, occupation, location of residence, and pregnancy period

^d^OBGYN, obstetrics and gynecology

^e^Adjusted for maternal age, education, occupation, location of residence, and other covariates

### Barriers against influenza vaccination and factors associated with future vaccination

Among 192 women in the unvaccinated group, 169 (88%) responded to the question about reasons for not receiving the influenza vaccine (“Why did you not receive the influenza vaccination?”). Fifty-nine (34.9%) respondents replied, “I did not know if I should be vaccinated,” and 37 (21.9%) answered, “I am planning to be vaccinated according to the appropriate vaccination schedule.” Seventy-three (43.2%) respondents replied, “I did not want to have the influenza vaccination.” Participants who responded that they did not want to be vaccinated were asked their reason, and all respondents (n = 73) answered that they “did not know the importance of vaccination” (Table 3).

**Table 3:** Reasons for not wanting an influenza vaccination in unvaccinated women and factors for future vaccination during pregnancy

Reasons for not wanting influenza vaccination in unvaccinated women (multiple responses) (Total responders, *n* = 73)	
Not knowing the importance of vaccine	48 (66%)
Not knowing the importance of vaccine + Distrust of effect	1 (1%)
Not knowing the importance of vaccine + Fear of side effects	1 (1%)
Not knowing the importance of vaccine + Others	23 (32%)
Major influencing factors for future vaccination during pregnancy (multiple responses) (Total responders, n = 92)	
OBGYN^a^ doctors	81 (88%)
Other medical doctors^b^	4 (2.1%)
Family or friends	4 (2.1%)
TV/radio/paper/internet	9 (9.8%)
Free vaccination program	16 (17.4%)
Pediatric doctors	8 (8.7%)

^a^OBGYN, obstetrics and gynecology; ^b^Other medical doctors, medical doctors other than OBGYN or pediatric doctors

Among 192 women included in the unvaccinated group, 92 responded to the question about influencing factors for future vaccination, and multiple responses were allowed (“If you did not know the importance of vaccination, in which cases would you get the vaccination?”). The 92 women who responded provided 122 choices. The majority of the respondents (n = 81, 88%) said that they would get the influenza vaccine in the future if their obstetrician recommended it.

### Demographic characteristics, awareness, and attitudes of OBGYN doctors

A total of 373 questionnaires was eligible to be analyzed after excluding three incomplete questionnaires and 86 responses from non-OBGYN doctors. Questionnaires were collected through an online survey (88.7%, 331/373) or a paper survey (11.3%, 42/373). As shown in Fig. 1(B) and Suppl. 3, the distribution of OBGYN doctors’ clinics or hospitals suggests that 80.5% of respondents worked in metropolitan areas, with 37.8% in Seoul and 43.2% in Gyeonggi and other metropolitan cities. Demographic characteristics, awareness, and attitudes are presented in Table 4. A total of 287 (76.9%) of the 373 OBGYN doctors was included in the routine recommendation group. Significant differences were found between routine and passive recommendation groups, including 1) affiliation with a private clinic or hospital; 2) personal influenza vaccination in the previous year; 3) provided maternity care within the last five years; 4) awareness of KCDC guidelines; 5) agreement with the recommendations for influenza vaccine during pregnancy; 6) appropriate time for influenza vaccination during pregnancy; and 7) awareness of the 2019 national free influenza vaccination program for pregnant women.

**Table 4:** Demographic characteristics, awareness, and attitudes of OBGYN^a^ doctors associated with recommendations of maternal influenza vaccination

Characteristics	Routine recommendation group (n = 287)	Passive recommendation group (n = 86)	p-value
Age (years) (mean ± SD)	47.51 ± 9.4	45.76 ± 12.52	0.231
Female (n, %)	154 (53.7)	54 (62.8)	0.135
Private clinic/hospital (n, %)	263 (91.6)	54 (62.8)	< 0.001
Metropolitan area (n, %)	231 (80.5)	71 (82.6)	0.668
Provided maternity care within the last 5 years (n, %)	259 (90.2)	68 (79.1)	0.006
Received influenza vaccination in the previous year (n, %)	272 (94.8)	71 (82.6)	< 0.001
Awareness of safety, importance, and priority groups of vaccination before, during, and after delivery, recommended by KCDC^b^ guidelines (n, %)	278 (96.9)	68 (79.1)	< 0.001
Do you provide information about influenza vaccine to pregnant women?			< 0.001
Always (n, %) Sometimes (n, %) No (n, %)	276 (96.2) 11 (3.8) 0 (0)	8 (9.3) 61 (70.9) 17 (19.8)	
Appropriate time for influenza vaccination related to pregnancy^c^ (n, %) All trimesters, pre-pregnancy, and postpartum 2^nd^ and 3^rd^ trimesters, pre-pregnancy, and postpartum 2^nd^ and 3^rd^ trimesters and postpartum	158 (70.2%) 61 (27.1%) 6 (2.7%)	33 (49.3%) 26 (38.8%) 8 (11.9%)	0.001
Awareness about 2019 pregnant women free vaccination (n, %)	268 (93.4)	51 (59.3)	< 0.001

^a^OBGYN, obstetrics and gynecology; ^b^KCDC, Korean Centers for Disease Control and Prevention; ^c^A total of 292 OBGYN doctors responded to this question

### Influencing factors for influenza vaccine recommendation by OBGYN doctors

In univariate analysis, “working at a private clinic or hospital”, “provided maternity care within the last 5 years”, “received influenza vaccination in the previous year”, “awareness of KCDC guidelines”, and “awareness of the 2019 national free influenza vaccination program” were significantly associated with routine vaccine recommendation by OBGYN doctors (Table **5**). In a multivariate analysis, “working at a private clinic or hospital” (OR 5.33, 95% CI 2.44–11.65), “awareness of KCDC guidelines” (OR 3.11, 95% CI 1.11–8.73), and “awareness of the 2019 national free influenza vaccination program for pregnant women” (OR 4.88, 95% CI 2.34–10.17) were associated with routine recommendation**.** However, “provided maternity care within the last 5 years” and “received influenza vaccination in the previous year” were not significant in the multivariate model.

**Table 5:** Associated factors for routine vaccine recommendation among respondent OBGYN^a^ doctors

Factors	Univariate analysis					Multivariate analysis			
	**Crude OR**^**b**^		**95% CI**^**c**^	**p-value**		**Adjusted OR**^**e**^		**95% CI**	**p-value**
Work place									
Public office/hospital	1				1				
Private clinic/hospital	6.49	3.55–11.89		< 0.001	5.33		2.44–11.65		< 0.001
Provided maternity care within the last 5 years									
No	1				1				
Yes	2.45	1.28–4.69		0.007	1.66		0.74–3.70		0.216
Received influenza vaccination in the previous year									
No	1				1				
Yes	3.83	1.79–8.21		0.001	2.56		0.96–6.81		0.061
Awareness of safety, importance, and priority groups of vaccination before, during, and after delivery, as recommended by KCDC^d^ guidelines									
No	1				1				
Yes	8.18	3.52–18.99		< 0.001	3.11		1.11–8.73		0.031
Awareness about 2019 national free influenza vaccination program									
No	1				1				
Yes	9.68	5.14–18.24		< 0.001	4.88		2.34–10.17		< 0.001

^a^OBGYN, obstetrics and gynecology; ^b^OR, odds ratio; ^c^CI, confidence interval; ^d^KCDC, Korean Centers for Disease Control and Prevention

^e^Adjusted for age, sex, and location of clinic or hospital, together with covariates

### Factors expected to effect OBGYN future recommendations for maternal influenza vaccination (multiple responses)

Among 235 choices from 217 respondents, guidelines recommended by the government or public health (108, 46%) and academic committees (59, 25%) were endorsed as major factors influencing OBGYN doctors’ future recommendations for maternal influenza vaccination. Other factors included academic papers and lectures (31, 13%), media advertisements (28, 12%), and free vaccination (9, 4%).

## Discussion

In this study, 63.2% of respondent pregnant women were vaccinated against influenza, and 76.9% of respondent OBGYN doctors routinely recommended the influenza vaccine for pregnant women. Univariate and multivariate analyses showed that having ever received information about influenza vaccination during pregnancy, especially from OBGYN doctors, and second/third trimester were associated with influenza vaccination in pregnant women. The most significant barrier to influenza vaccination among pregnant women was a lack of awareness. In the survey for OBGYN doctors, independent factors effecting routine recommendation were working at a private clinic or hospital, awareness of KCDC guidelines, and awareness of the national free influenza vaccination program for pregnant women during the 2019 flu season.

Pregnancy-related vaccination rates in the US were between 49.1% and 53.6% from 2015 to 2018 [19]. In Ireland, the highest vaccination rates reported in pregnant women were 62% and 58% during the 2017–18 and 2016–2017 flu seasons, respectively [20]. The highest vaccination rate during pregnancy in Western Australia was 61% in 2015 [21]. Previous questionnaire studies from Korea reported 35%–40% vaccination rates in pregnant women [14, 15]. However, vaccination coverage for the total pregnant population of Korea was unknown until the 2018–2019 flu season because influenza vaccination was not covered by insurance and was performed in private settings. We speculate that the national free influenza vaccination program, which was initiated for the 2019–2020 flu season in Korea, might have increased awareness of vaccination and confidence among OBGYN doctors, which could lead to enhanced routine recommendations for pregnant women. A previous study of Korean obstetricians about maternal influenza vaccination reported that only 26.5% of obstetricians strongly recommended maternal influenza vaccination [22]. It is well known that advice and encouragement from familiar healthcare professionals (HCPs) significantly improve vaccine acceptance in pregnant women [23]. Additionally, several studies have shown that HCP knowledge about vaccine efficacy and safety is significantly associated with their vaccine recommendations. HCP confidence about vaccination is crucial for vaccination implementation in pregnant women [24–26]. Because OBGYN doctors are most familiar with the conditions of pregnant women, the professional information and recommendations they provide can affect directly vaccination decisions. Because most of the OBGYN doctors in the present study considered vaccination guidelines an important factor for future recommendations, further education on existing guidelines and supporting position statements or programs by academic committees, especially those related to maternal–fetal medicine, could increase routine recommendations by OBGYN doctors by increasing confidence in their recommendations. Previous similar studies about maternal influenza vaccination with influencing factors were performed in only a few centers [15–19], so it is important to note that this study was performed across all provinces of Korea.

This study has several limitations. First, although this study included all provinces of Korea to maximize the demographic diversity of the study populations, it cannot be considered representative of all pregnant women and OBGYN doctors in Korea. Second, responding doctors and hospitals might have had positive attitudes toward vaccination, introducing the possibility of a selection bias, which could explain why our sample had nearly double the proportion of maternal influenza vaccination than that observed in previous Korean studies. Third, self-reported vaccination status might have introduced a potential reporting bias in our estimation. In addition, the number of non-respondents among the surveyed pregnant women and OBGYN doctors was not recorded. Finally, the cross-sectional nature of this study, rather than a prospective study, can be a limitation.

This study also has several strengths. First, our study included a significant number of respondents who lived or worked not only in metropolitan areas, but also respondents who lived or worked in non-metropolitan areas. Although the proportion of respondents who lived in local provinces was only about 20%, it correlated with the percentage of live births in local provinces, which was about 30% of all births in Korea [27]. Among the pregnant respondents, there was no significant difference in terms of residential distribution between the vaccinated and unvaccinated groups. Also, the effect of physician workplace location on routine versus passive recommendation was not significant. Second, the surveys of pregnant or postpartum women and OBGYN doctors were conducted anonymously, allowing free expression of opinions by the respondents. Most importantly, our study found a more than twice as large of a proportion of OBGYN doctors who routinely recommend maternal influenza vaccination compared with those in previous similar studies, which was correlated with a significantly increased proportion of maternal influenza vaccination.

## Conclusion

In this study, the proportion of pregnant women who reported receiving an influenza vaccination was 63.2%, and the proportion of OBGYN doctors who routinely recommended influenza vaccination was 76.9%. This study showed that providing information about maternal influenza vaccination, especially by OBGYN doctors, is crucial for increasing vaccination coverage in pregnant women. The 2019 national free influenza vaccination program in Korea facilitated recommendations of maternal influenza vaccination by OBGYN doctors. Based on the high acceptance rates for preventive vaccines in Korea [28, 29], the maternal influenza vaccination program could be more successful with provision of the appropriate information. Close cooperation between the KCDC and OBGYN academic societies is crucial for enhancing the confidence of OBGYN doctors and their recommendations to increase influenza vaccination during pregnancy, which will maximize the benefits of the vaccine for both mothers and infants.

## Supplementary Information

**Additional file 1.** Questionnaire. Survey of pregnant or postpartum women: vaccination during pregnancy.

**Additional file 2.** Questionnaire. Survey of physicians: vaccination during pregnancy.

**Additional file 3.** Table and figure. Detailed geographical distributions of respondents.

## Data Availability

Derived data supporting the findings of this study are available from the corresponding author on request.

## Abbreviations

OBGYN: Obstetrics and gynecology
OECD: Organization for Economic Cooperation and Development
WHO: World Health Organization
KCDC: Korean Centers for Disease Control and Prevention
KSMFM: Korean society of Maternal–Fetal Medicine
KSPM: Korean Society of Perinatal Medicine
KAOG: Korean Association of Obstetrics and Gynecology
KSUOG: Korean Society of Ultrasound in Obstetrics and Gynecology
HCP: Healthcare professional
