# Supplementary material for: Influenza vaccination during pregnancy and influencing factors in Korea: A multicenter questionnaire study of pregnant women and obstetrics and gynecology doctors
Source: BMC Pregnancy Childbirth. 2021 Jul 16;21:511. doi: 10.1186/s12884-021-03984-2 (PMC8285826; doi:10.1186/s12884-021-03984-2)
Supplement: Supplementary file 1 — Additional file 1. Questionnaire. Survey of pregnant or postpartum women: vaccination during pregnancy. [file 12884_2021_3984_MOESM1_ESM.docx]

**S1 questionnaire. Survey of pregnant or postpartum women: vaccination during pregnancy**

1. Age: ____ years old
2. Gestational or postpartum weeks and conception method

Gestational weeks: ___________weeks

Postpartum: ___________weeks

Assisted reproduction: ☐ Yes☐ No

3. Previous delivery history

☐ Yes __________times (last delivery year: _________)

☐ No

4. Place of residence

☐ Seoul ☐ Gyeonggi☐ Chungcheong☐Gyeongsang☐Jeolla☐Gangwon☐Jeju

☐ Metropolitan cities of Busan, Incheon, Kwangju, Daegu, Ulsan, Daejeon, or Sejong

5. Occupation

☐ Housewife

☐ Employed, but not related to health care, including self-employed, in-service, office workers engaged in education, production, or study

☐ Healthcare professionals (medical doctor, nurse, midwife)

☐ Others: ____________

6. Highest degree completed:

☐ Primary/Middle/High school

☐ Bachelor/University college/Master/University

7. Have you ever received information about influenza vaccination during pregnancy?

☐ Yes

☐ No

7-1. **If yes**, from whom did you receive that information?

- OBGYN (obstetrics and gynecology) doctor
- Pediatric Doctor
- Medical doctors (except obstetrics and gynecology doctor or pediatric doctor)
- Public health office
- Media (television, radio, internet, and so on)
- Family, friend, or acquaintance
- Others____________

1. Have you received influenza vaccination during pregnancy in the 2019-2020 flu season?

☐ Yes, then administered place: ☐public health care office ☐ clinic or hospital

☐ No

9. (For women with delivery history) Did you receive influenza vaccination in the previous pregnancy?

☐ Yes

☐ No

10. If you have not received influenza vaccination, what is the reason?

- - - - 1. Because I didn’t want to have influenza vaccination.
        2. Because I didn’t know if I should be vaccinated.

C. I’m planning to have vaccination at the time of vaccination schedule, but waiting for the appropriate time’

1. Others ________________________________

10-1. (**If question 10 answer A**) Why do you not wanting influenza vaccination? (Multiple responses available)

- Not knowing the importance of vaccine
- Fear of pain
- Distrust of effect
- Fear of side effect for fetus
- Fear of side effects for myself
- Burden of cost
- Religious belief
- Simply forgot to take vaccination
- Had negative information about influenza vaccination from media
- Lack of enough information to make a decision to have influenza vaccination
- Others _________________________________

10-2. (**If question 10 answer B**) If you did not know the importance of vaccination, in which case would you get the vaccination? (Multiple responses available)

- Recommendation from maternity HCPs
- Recommendation from General HCPs (other than maternity HCP or pediatric HCPs)
- Recommendation from pediatric HCPs
- Recommendation from family of friends
- Recommendation from TV/Radio/Paper/internet
- Free vaccination program
