# Supplementary material for: Influenza vaccination during pregnancy and influencing factors in Korea: A multicenter questionnaire study of pregnant women and obstetrics and gynecology doctors
Source: BMC Pregnancy Childbirth. 2021 Jul 16;21:511. doi: 10.1186/s12884-021-03984-2 (PMC8285826; doi:10.1186/s12884-021-03984-2)
Supplement: Supplementary file 2 — Additional file 2. Questionnaire. Survey of physicians: vaccination during pregnancy. [file 12884_2021_3984_MOESM2_ESM.docx]

**S2 questionnaire. Survey of Physicians: vaccination during pregnancy**

1. Sex ☐ Male ☐ Female
2. Age _______ years old
3. When was your last time you provided maternity care?

☐ Within 5 years ☐ More than 5 years ☐ Others _____________

1. What medical specialty do you have?

☐ Obstetrics and gynecology ☐ Pediatrics

☐ General physician or other specialties (Not obstetrics and gynecology nor pediatrics)

1. In which level of hospital/clinic do you practice?

☐ Tertiary hospital (Public hospital)

☐ Tertiary hospital (private hospital )

☐ Secondary hospital (Public hospital)

☐ Secondary hospital (private hospital)

☐ Primary hospital

☐ Private clinic

1. Located administrative district of your working place?

☐ Seoul ☐ Gyeonggi ☐ Chungcheong ☐ Gyeongsang ☐ Jeolla ☐ Gangwon ☐ Jeju

☐ Metropolitan cities of Busan, Incheon, Kwangju, Daegu, Ulsan, Daejeon, or Sejong

7. Did you aware of KCDC guideline ‘all pregnant or breastfeeding women during flu season are primarily recommended to receive an inactivated influenza vaccination, regardless of trimester of pregnancy’?

☐ yes ☐ No

8. What is your opinion on recommending influenza vaccination during pregnancy?

☐ Strongly agree ☐ Somewhat agree ☐ Neither Agree nor disagree

☐ Somewhat disagree ☐ Disagree

9. Do you provide information about influenza vaccination in pregnant women?

☐ Always provide information about influenza vaccination

☐ Sometimes provide information about influenza vaccination

☐ Never provide information about influenza vaccination

9-1. (If question 14, answer ‘Never’) If not, what was the reason you doesn’t provide the information?

☐ Because I don’t think it is necessary

☐ Lack of explanatory material (Pamphlet, Poster)

☐ Lack of time to explain

☐ Lack of information about influenza vaccination

☐ Others : ______________

10. Do you recommend influenza vaccination in pregnant women?

☐ Always recommend information about influenza vaccination

☐ Sometimes recommend information about influenza vaccination

☐ Never recommend information about influenza vaccination

10-1. (If question 10, answer ‘always’ or ‘sometimes’) When would be the appropriate time for influenza vaccination?

☐ 1^st^, 2^nd^, 3^rd^ trimester, preparing pregnancy, and postpartum (within 6 weeks)

☐ 2^nd^, 3^rd^ trimester, preparing pregnancy, and postpartum (within 6 weeks)

☐ 2^nd^, 3^rd^ trimester, postpartum women (within 6 weeks)

10-2. (If question 10, answer ‘never’) If you never recommend influenza vaccination, what is the reason?

☐ Because I didn’t know that influenza vaccination is recommended during pregnancy.

☐ I don’t agree recommending influenza vaccination during pregnancy

-> what is the reason?

☐ uncertain about effect ☐ concern of side effect
☐ others___________________

☐ Reasons of patient

☐ Others:

11. Did you get influenza vaccination previous flu season (2018-2019)?

☐ Yes ☐ No

12. What factors do you expect to affect the future recommendation of influenza vaccination in pregnant women? (Multiple responses available)

☐ The guidelines from government or public healthcare

☐ The guidelines from academic committee

☐ Academic papers about influenza vaccination

☐ advertisement of media (TV/Radio/Internet)

☐ Academic lectures about influenza vaccination

☐ Free vaccination program

13. Did you recognize that pregnant women were included in Free vaccination program from 2019 fall?

☐ Yes ☐ No
