# Supplementary material for: Influenza vaccination during pregnancy and influencing factors in Korea: A multicenter questionnaire study of pregnant women and obstetrics and gynecology doctors
Source: BMC Pregnancy Childbirth. 2021 Jul 16;21:511. doi: 10.1186/s12884-021-03984-2 (PMC8285826; doi:10.1186/s12884-021-03984-2)
Supplement: Supplementary file 3 — Additional file 3. Table and figure. Detailed geographical distributions of respondents. [file 12884_2021_3984_MOESM3_ESM.docx]

|  | **Pregnant/Postpartum women** | **OBGYN doctors’ clinics/hospitals** |
| --- | --- | --- |
| **Metropolitan cities** | N (%) | N (%) |
| Seoul | 236 (45.2%) | 141(37.8%) |
| Gyeonggi | 124 (23.8%) | 83(22.3%) |
| Other cities (Incheon, Daejeon,   Gwangju, Busan, Ulsan, Daegu) | 62 (11.9%) | 78(20.9%) |
| **Non-metropolitan cities** |  |  |
| Chungcheong | 9 (1.7%) | 24(6.4%) |
| Gyeongsang | 10 (1.9%) | 28(7.5%) |
| Jeolla | 19 (3.6%) | 8(2.1%) |
| Gangwon | 23 (4.4%) | 4(1.1%) |
| Jeju | 39 (7.5%) | 7(1.9%) |

S5. Geographical distributions of respondents


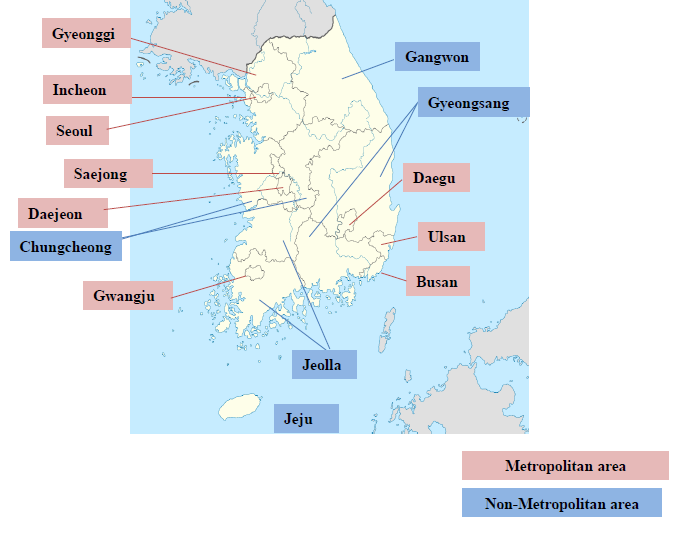
This map is edited from the map in Wikimedia Commons.
